# A cohesive multidisciplinary team approach to the management of patients with eating disorders

**DOI:** 10.1186/2050-2974-1-S1-O15

**Published:** 2013-11-14

**Authors:** Sarah Braham, Kate Sampson

**Affiliations:** 1 Robina Hospital, Australia

## 

Eating disorders can be a life threatening illness not only due to the effects of starvation, but also the effects of impaired capacity. At Robina Hospital, patients admitted with a BMI < 14 are admitted to the Acute Medical Unit (AMU) for nutritional restoration and medical management before being transferred to a suitable mental health ward for ongoing treatment.

The AMU at Robina has been managing patients with eating disorders, in particular Anorexia Nervosa for a number of years. The treatment plan implemented by the Acute Medical team is considered to be of a very high standard by the Eating Disorders Outreach Service (EDOS), a specialist consulting liaison service provided by the Royal Brisbane and Women's Hospital. The AMU prides itself on developing a cohesive multidisciplinary team approach, striving for best practice management by utilising protocols and guidelines developed by the EDOS service.

This presentation will discuss the experiences of the Acute Medical Team in managing eating disorder patients. It will outline the team structure, current practices and guidelines used and identify areas of improvement recognised through lessons learned.

The aim is to present information and experiences that may assist other acute inpatient medical settings in managing eating disorder patients discussing evidence based strategies developed to support and improve the management and physical health of patients with eating disorders.

This abstract was presented in the **Care in Inpatient and Community Settings** stream of the 2013 ANZAED Conference.

